# Multi-Scale Characean Experimental System: From Electrophysiology of Membrane Transporters to Cell-to-Cell Connectivity, Cytoplasmic Streaming and Auxin Metabolism

**DOI:** 10.3389/fpls.2016.01052

**Published:** 2016-07-25

**Authors:** Mary J. Beilby

**Affiliations:** School of Physics, The University of New South Wales, SydneyNSW, Australia

**Keywords:** Characeae, cell-to-cell transport, cytoplasmic droplets, cytoplasmic streaming, metabolic pathways, plasma membrane transporters, plasmodesmata, tonoplast transporters

## Abstract

The morphology of characean algae could be mistaken for a higher plant: stem-like axes with leaf-like branchlets anchored in the soil by root-like rhizoids. However, all of these structures are made up of giant multinucleate cells separated by multicellular nodal complexes. The excised internodal cells survive long enough for the nodes to give rise to new thallus. The size of the internodes and their thick cytoplasmic layer minimize impalement injury and allow specific micro-electrode placement. The cell structure can be manipulated by centrifugation, perfusion of cell contents or creation of cytoplasmic droplets, allowing access to both vacuolar and cytoplasmic compartments and both sides of the cell membranes. Thousands of electrical measurements on intact or altered cells and cytoplasmic droplets laid down basis to modern plant electrophysiology. Furthermore, the giant internodal cells and whole thalli facilitate research into many other plant properties. As nutrients have to be transported from rhizoids to growing parts of the thallus and hormonal signals need to pass from cell to cell, Characeae possess very fast cytoplasmic streaming. The mechanism was resolved in the characean model. Plasmodesmata between the internodal cells and nodal complexes facilitate transport of ions, nutrients and photosynthates across the nodes. The internal structure was found to be similar to those of higher plants. Recent experiments suggest a strong circadian influence on metabolic pathways producing indole-3-acetic acid (IAA) and serotonin/melatonin. The review will discuss the impact of the characean models arising from fragments of cells, single cells, cell-to-cell transport or whole thalli on understanding of plant evolution and physiology.

## Introduction

**FIGURE 1:**
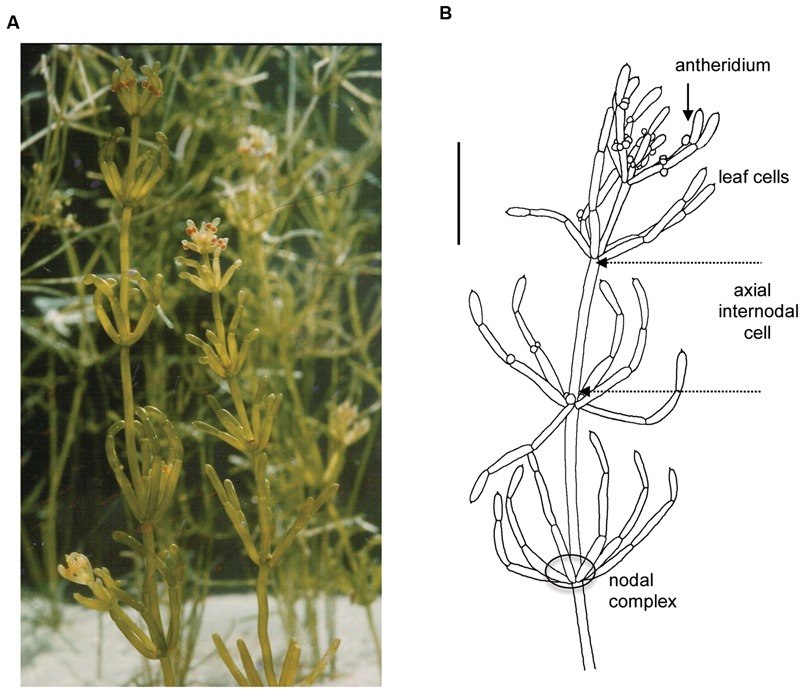
**General morphology of the characean plant.** *Chara australis* is one of the most used characean experimental systems. **(A)** Plants are perennial and easily cultured in the lab setting for many years. **(B)** Male plant, identified by orange antheridia, with whorls of six branchelets. Multicellular nodes connect the axial internodes and the branchelets. The rhizoids (not shown) are also made up of large cells joined end to end. The scale bar is 10 mm. Part (B) is adapted from Beilby and Casanova (2013). For more details of Characeae morphology and variation between species see Beilby and Casanova (2013).

From all the charophytes, Characeae morphology appears most similar to embryophytes (land plants). The thallus consists of axial stem with leaf-like side branches and is anchored in the soil by root-like rhizoids. However, all these structures are made from large single cells with multiple nuclei, connected by nodal complexes consisting from small cells with single nuclei (**Figure 1**). The axial internode cells grow up to diameter of 1 mm and up to half meter long in some species (see chapter 1 of Beilby and Casanova, 2013). New thalli regenerate from the nodal complexes.

Initially, Characeae seemed to be the closest streptophyte algal relatives to land plants (Karol et al., 2001), but recently Coleochaetophyceae or Zygnematophyceae moved into that position (Wodniok et al., 2011; Timme et al., 2012). Wickett et al. (2014) provide strong support for Zygnematophyceae to be the sister-group to land plants. This result is confirmed by plastid phylogenomics (Ruhfel et al., 2014) and plastid genome content (de Vries et al., 2016). Now that *Chara braunii* genome is in process of being sequenced and annotated and sequencing of the members from the other two classes is imminent, we can look forward to more complete solution to this puzzle.

The size of characean internodal cells makes it a good system for electrophysiology (for review see chapters 2 and 3 of Beilby and Casanova, 2013; Beilby, 2015). These cells fully recover after excision from the thallus and can be subjected to prolonged experiments (24 h and more) with multiple electrodes. For history of pioneering electrical and transport measurements on the characean plants see Walker (1955) and Hope and Walker (1975).

In this review, I will touch on electrophysiology, but mainly in context of exploring transporters at the tonoplast and plasma membrane often initially described in the Characeae. Some of these transporters contribute to supplying the plant with chloride, nitrogen, phosphorus and potassium – elements vital to all plants (carbon transport is covered in another review: Beilby and Bisson, 2012). The movement of ions and nutrients through the characean thallus depends strongly on cytoplasmic streaming. The characean cell size and morphology facilitated the identification of the streaming mechanism. Plasmodesmata are another important element of cell-to-cell transport with some basic experiments made possible by the characean cell size and organization. Another aspect of plant physiology that can be studied in Characeae is metabolic pathways. Beilby et al. (2015) found circadian changes in endogenous concentration of indole-3-acetic acid (IAA) in *Chara australis*, confirming that the entanglement of this important hormone with the circadian clock pre-dates the emergence of plants on land.

## One Plant – Many Experimental Systems

The morphology of Characeae facilitates creation of experimental systems on many different levels:

(i) Cytoplasmic droplets that allow patch clamp investigation of tonoplast channels.
(ii) Perfusion and permeabilization that create access to both sides of plasma membrane and tonoplast in single cell context.
(iii) Multi-compartmented cell holders that expose different parts of single cell to different media and tracer substances.
(iv) Two or more tandem cells to measure cell-to-cell transport electrically and by tracers.
(v) Whole thallus assays, where the results are dominated by contents of the large axial and leaf internodes with similar biochemistry and structure.

### Perfusion, Permeabilized Plasma Membrane and Cytoplasmic Droplets: Tonoplast Transporters

**FIGURE 2:**
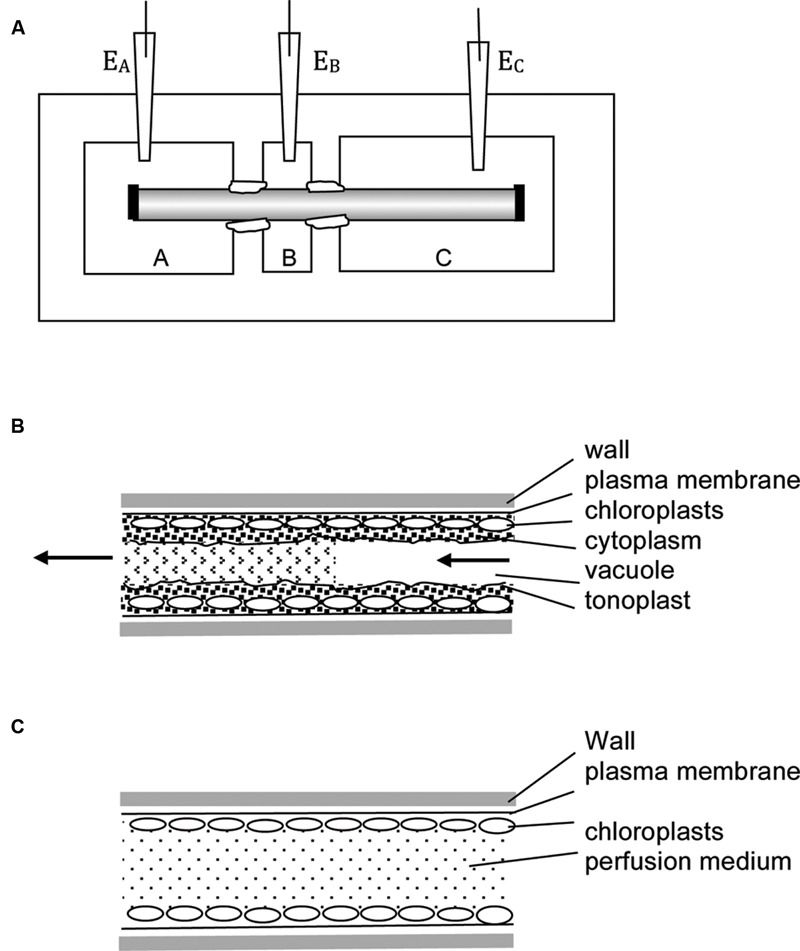
**Experimental techniques. (A)** Multi-compartment Perspex or Lucite cell holder is employed in many types of experiments. The axial internodes grow to up to 30 cm, so more than three compartments can be introduced. The compartments are electrically insulated by applying silicon grease or Vaseline to the cells at each partition. In the three-compartment holder one compartment (e.g., B) can be filled with 50 – 100 mM KCl, reducing the membrane PD to zero due to activation of high conductance K^+^ channels (Beilby, 1985). The other compartments are filled with artificial pond water (APW) with osmolarity adjusted, so no water transport occurs between parts of the cell. The reading of electrodes E_A_ – E_B_ approximates the trans-membrane PD. In this technique no electrode insertion is necessary – very useful for wound and mechanical stress investigations. To perform vacuolar or cytoplasmic perfusion, chambers A and C are filled with perfusion medium, B with external medium. The nodes at the ends of the cell (shown as black rectangles) are cut and pressure gradient introduced, so that the vacuolar medium is replaced by artificial medium. In this case electrode E_A_ or E_C_ become the “internal” electrodes, while electrode E_B_ is the external electrode. The rate of perfusion and/or the perfusion medium composition determines if the cell retains tonoplast **(B)** or not **(C).** For more details see text.

The cylindrical symmetry of the large internodal cells allows perfusion of the vacuolar contents. The cell is put in a three-well holder (**Figure 2A**) with perfusion medium in pools A and C and the cell ends are cut. An inclination of the cell holder or higher fluid level in one of the wells creates pressure gradient to replace the vacuolar sap by artificial medium (**Figure 2B**, for the detailed technique description see Tazawa, 1964; Beilby, 1989). The perfusion can be repeated with different media, but cells are not turgid and survive only some hours. For electrical contact, the “internal electrode” is submerged into one of the outer compartments, and the “external electrode” into the middle compartment. The cell ends can be ligated with thread to ensure longer survival, but the composition of the medium changes with time.

#### Tonoplast Proton Pumps

**FIGURE 3:**
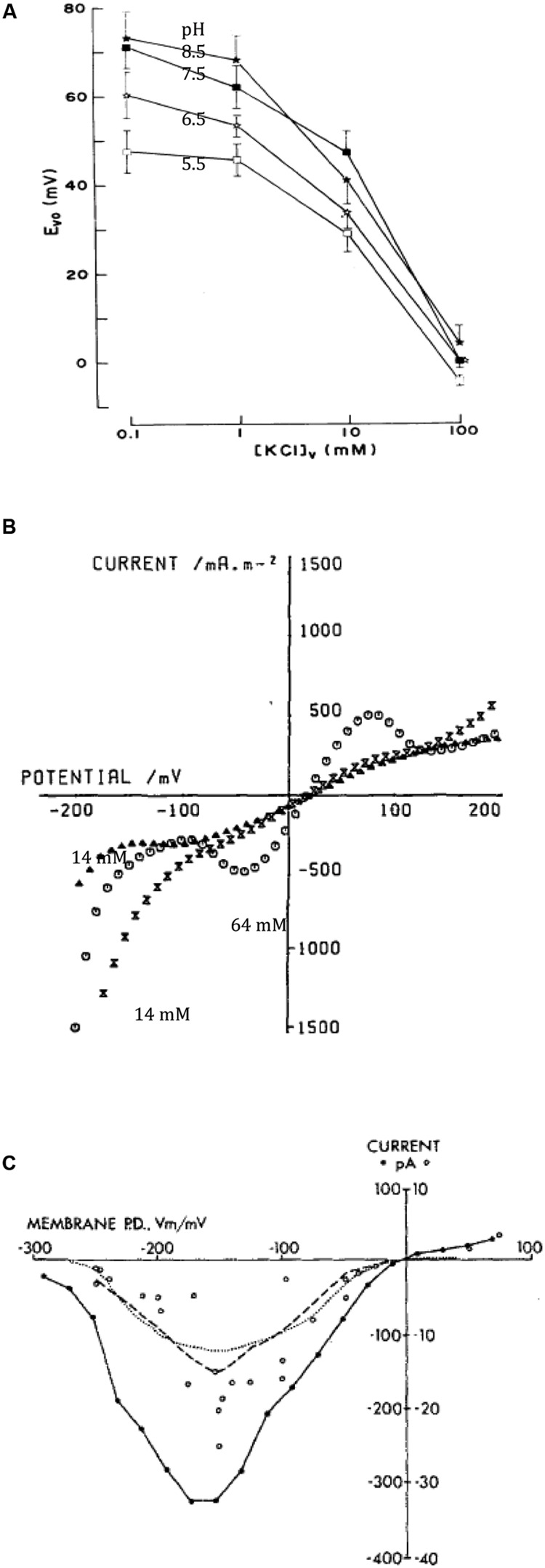
**Tonoplast transporters. (A)** Vacuolar PD (E_vo_) as function of vacuolar perfusion medium K^+^ concentration and pH (for details see Moriyasu et al., 1984b). **(B)** I/V characteristics of the tonoplast with natural vacuolar sap and permeabilized plasma membrane with high (64 mM, empty circles) or low (14 mM, triangles before high concentration exposure and crossed triangles after) cytoplasmic Cl^-^ (Tester et al., 1987). **(C)** Time-averaged I/V characteristics of single tonoplast K^+^ channels from several patches from cytoplasmic droplets (Laver and Walker, 1987).

Moriyasu et al. (1984a) demonstrated that the vacuolar pH is regulated close to 5, regardless of pH of the external medium. The perfused cells responded slowly to pH increase with inhibition by Dicyclohexylcarbodiimide (DCCD), while pH decrease was corrected quickly without inhibition, suggesting presence of ATP powered proton pump as well as H^+^/OH^-^ channels in the tonoplast. To resolve tonoplast electrical characteristics Moriyasu et al. (1984b) increased the conductance of the plasma membrane by including 110 mM KCl in the external medium, opening large conductance K^+^ channels. The potential difference (PD) across the tonoplast then dominated the combined PD across both membranes. Changing K^+^ concentration in the perfusion medium revealed passive tonoplast PD due to potassium (**Figure 3A**). At low internal K^+^ (0.1 mM) the PD generated by the proton pump, while regulating vacuolar pH, could be measured and increased up to +30 mV at high vacuolar pH (**Figure 3A**). As K^+^ concentration in the perfusion medium approached the level of the sap (∼100 mM), the pump PD was short-circuited (**Figure 3A**). So, there is an interesting difference between the two membranes: the plasma membrane H^+^ ATPase shuts down, when the membrane conductance becomes dominated by K^+^ channels (Beilby, 1985), while the tonoplast proton pump/s work against high K^+^ conductance background with high K^+^ concentrations (∼100 mM) in the cytoplasm and vacuole.

Shimmen and MacRobbie (1987) employed the technique of permeabilization to disintegrate the plasma membrane by removing external and wall Ca^2+^ by ethylene glycol tetraacetic acid (EGTA). While the chloroplasts were disrupted, the tonoplast and the vacuolar compartment were largely unchanged. The cells were exposed to medium simulating the main features of the native cytoplasm (Shimmen and Tazawa, 1982) and ATP or Pyrophosphate (PPi) concentrations could be controlled. The cells were perfused and their ends ligated prior to permeabilization to control the vacuolar medium and to eliminate its buffering capacity. The H^+^ pumping ability of the tonoplast was judged by accumulation of neutral red in the cell constructs. Shimmen and MacRobbie (1987) found two distinct types of proton pump in the tonoplast, energized either by ATP or PPi. The PPase needs K^+^ and Mg^2+^, does not respond to ${\mathrm{NO}}_{3}^{–}$ and is less affected by DCCD. The ATPase needs Mg^2+^ but not K^+^, ${\mathrm{NO}}_{3}^{–}$ applied from the cytoplasmic side inhibits its activity and DCCD is a more powerful inhibitor. Both tonoplast pumps can be distinguished from the plasma membrane ATPase, as they are not affected by cytoplasmic vanadate.

#### I/V Characteristics of Pumps and Cl^-^ Channel

Tester et al. (1987) permeabilized young *Chara* leaf cells and measured I/V characteristics between -200 and +200 mV. With low cytoplasmic Cl^-^, the sigmoid profile crossed the axis at slightly positive PD. A rise in cytoplasmic Cl^-^ concentration from 14 to 64 mM increased the conductance in PD-dependent manner, suggesting opening of Cl^-^ channels (**Figure 3B**). The equilibrium PD is positive in this preparation, as the reference electrode was placed in the cytoplasmic phase and the internal electrode in the vacuole. A drop in cytoplasmic K^+^ concentration from 113 to 30 mM increased the tonoplast conductance in several experiments, somewhat contradictory to results of Moriyasu et al. (1984b).

#### K^+^ Channels

To utilize new (then) technique of patch clamping, cytoplasmic droplet technique was developed by Luhring (1986) using results from Moriyasu et al. (1984a). A cut end of a slightly flaccid internodal cell was immersed in the vacuolar sap-like medium, producing droplets, which did not form cell walls. Sakano and Tazawa (1986) demonstrated the vacuolar origin of the membrane by fluorescence after perfusion with Concanavalin A/fluorescein isothiocyanate (FITC). The droplets were very stable and accessible to patch-clamp electrodes in both drop-attached and excised configurations.

Luhring (1986), Laver and Walker (1987) and Laver et al. (1989) made detailed studies of the conductive (170 pS) K^+^ channel in the droplets. Laver and Walker (1987) formulated mathematical model with one fully open state and seven closed states. The average I/V characteristics exhibited a maximum between -100 and -200 mV, somewhat puzzling result, as tonoplast PD is usually positive (**Figure 3C**). Laver and Walker (1991) described channel activation by cytoplasmic Ca^2+^ concentration from 0.1 – 1 μM, with three bound calcium ions necessary for opening. However, for cytoplasmic Ca^2+^ concentrations above 10 μM, Ca^2+^ could act as a block. Laver (1992) distinguished two binding sites for Ca^2+^ in the vestibule to the vacuole and three binding sites for Ca^2+^ on the cytoplasmic side. Laver et al. (1997) observed that calmodulin inhibitors W-7 and trifluoperazine (TFP) affected the channel open state, with TFP promoting a new sub-state, but the channel was not Ca^2+^-calmodulin activated. Homble and Fuks (1991) observed partial block by tetraethylammonium (TEA) on either side of the membrane. Bertl (1989) replaced K^+^ by Na^+^, blocking the channel on either side of the tonoplast. The addition of Na^+^ to K^+^ resulted in regions of negative conductance. Tyerman et al. (1992) resolved conductance sub-states: a longer residency located near the main open state, while a “mid-state” occurred after fast transitions from the main state. Draber et al. (1993) and Schultze and Draber (1993) detected spontaneous cooperative behavior of K^+^ channels that might arise from channel clustering in the membrane. Katsuhara et al. (1989) suggested that Ca^2+^-dependent K^+^ currents across the tonoplast have an important role in hypotonic regulation in salt tolerant *Lamprothamnium succinctum*.

Another type of K^+^ channel with smaller conductance (∼90 pS) was also observed in tonoplast droplets (Tyerman and Findlay, 1989; Pottosin and Andjus, 1994). Pottosin and Andjus (1994) patch-clamped droplets of *C. gymnophylla* and classified these channels as slow delayed rectifier, activated by depolarization, not high Ca^2+^ and blocked by TEA, and Cs^+^. The authors suggested a role for repolarization after excitation event.

#### Cl^-^ Channels

The Cl^-^ channels with a conductance of ∼ 21 pS were also detected in the droplets (Tyerman and Findlay, 1989). In droplet attached mode with media of 130 mM Cl^-^ outside and ∼15 mM Cl^-^ inside the channel behaved as an outward rectifier. The rectification disappeared in symmetrical Cl^-^ concentrations in detached patches. Berecki et al. (1999) measured channel activation by increased cytoplasmic (but not vacuolar) Ca^2+^ concentration. ZnCl_2_ (5 – 10 μM) acted as a block from cytoplasmic side. If the membrane PD was held negative of the reversal PD, larger negative currents were recorded, while pre-clamping to more positive PD produced larger positive currents. Low channel activity was observed at the normal cytoplasmic pH (7.2 – 7.4) with a half-maximal Ca^2+^ concentration of 100 – 200 μM (Berecki et al., 2001). At lower pH 6.0 the channel activity and mean open times became maximized at positive PDs and lower half-maximal activating Ca^2+^ concentration (5 μM), perhaps due to better calcium binding. Beilby et al. (1999) found more Cl^-^ channels in tonoplast of *Lamprothamnium* sp. as the external sulphated polysaccharide mucilage increased with cell age.

#### Comparison to Land Plant Tonoplast Transporters

The vacuolar ATPases were discovered in animal, fungi and land plant experimental systems. In land plants the proton pumping function was measured in late 1970 and early 1980s (see Beyenbach and Wieczorek, 2006 for historical account). The PPase was discovered in 1960s (see Hedrich and Schroeder, 1989 for review), but the proton pumping function was also realized later (Rea and Sanders, 1987). So, the elegant experiments of Moriyasu et al. (1984a,b) and Shimmen and MacRobbie (1987) confirmed results from isolated vacuoles and microsomes and placed it in evolutionary context. The sigmoid I/V characteristics observed for short periods in some experiments by Tester et al. (1987) may have been first recorded I/V characteristics of the vacuolar H^+^ pumps (compare **Figure 3B** with simulations from OnGuard model, Blatt et al., 2014). While in many of their experiments the ATP or PPi was not supplied in the permeabilizing medium, small amounts could have been retained near the vacuolar membrane. This experimental system clearly needs revisiting. Nakanishi et al. (1999) found the cDNA sequence of the *C. corallina* PPase 71% identical to that of land plants and 46% identical to that of chlorophyte *Acetabularia* and phototropic bacterium *Rhodospirillum rubrum*.

The vacuolar channels in land plants have been classified as slow activating (SV), fast activating (FV) and K^+^ selective (VK) (for review se Hedrich, 2012). The VK channel group includes two-pore channels (TPK). Patch clamp studies identified SV as non-selective cation channel, permeable to Na^+^ and under some conditions to Ca^2+^, which needs elevated cytoplasmic calcium level to open. In *Arabidopsis* genome TPC1 encodes the SV channel and loss of function mutants indicated that SV controls K^+^ homeostasis of the cell. Anion channels appear to be controlled by cell biochemistry and have been observed under elevated cytoplasmic calcium and in presence of kinases. CLC channels described in *Arabidopsis* genome operate as vacuolar anion channels or proton-Cl^-^ antiporters. Also in *Arabidopsis* the ALMT6 channels transport malate across the tonoplast. The SV channels seem to have appeared soon after plants came to land, as they were observed in patch clamp studies of *Physcomitrella patens* tonoplast (Koselski et al., 2013), but not in Characeae. In vascular land plants vacuoles have diversified to fulfill different functions in specialized tissues: for instance protein storage in a seed, storage of nitrogen in root tip, shrinking or swelling in stomata, storage of sulfate or storage of malate for crassulacean acid metabolism (CAM) photosynthesis (Martinoia et al., 2007). It will be interesting to compare sequences of K^+^, Cl^-^ and specialized channels in Characeae and land plants.

### Cytoplasmic Perfusion, Combination of Flux and Electric Current Measurement, Increasing the Size of Cytoplasmic Layer: Plasma Membrane Transporters

The tonoplast can be swept away by increasing the rate of perfusion flow or including EGTA in the medium (Williamson, 1975; Tazawa et al., 1976; **Figure 2C**). The experimenter now has access to both sides of the plasma membrane. The cell can be repeatedly perfused if the ends are left open. Alternatively, the ends are ligated and electrical contact is made by impaled electrode. In each case the cells are fragile and live only for some hours.

#### Cl^-^/H^+^ Symporter

**FIGURE 4:**
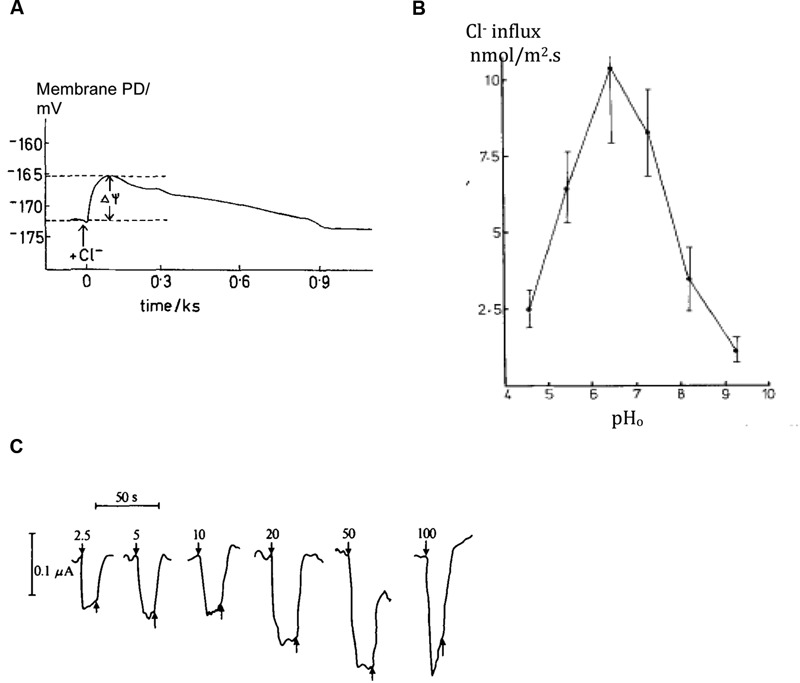
**Plasma membrane transporters: Cl^-^/H^+^ symporter.** **(A)** *Chara* cell Membrane PD depolarization upon re-supplying Cl^-^ after overnight starvation, see arrow (Sanders, 1980b) **(B)** Chloride influx in intact cells as function of external medium pH, pHo (Sanders, 1980b). **(C)** Positive inward currents observed in *Chara* cells with their resting PD voltage-clamped upon exposure to range of Cl^-^ concentrations shown next to each pulse in μM (Beilby and Walker, 1981).

With the negative membrane PD across the plasma membrane, Cl^-^ needs active transport into the cell even with the low concentration in the cytoplasm. Cells concentrate Cl^-^ in the vacuoles as osmoticum and to keep electroneutrality. Sanders (1980a) found an increase in Cl^-^ influx following period of Cl^-^ “starvation.” Membrane PD transiently depolarized upon resupply of Cl^-^ in the medium (**Figure 4A**). Using perfused tonoplast-less cells he established that this flux stimulation resulted from drop of cytoplasmic Cl^-^ concentration. He also observed a strong dependence of Cl^-^ influx on cytoplasmic and external pH (**Figure 4B**; Sanders, 1980b). The control of the cytoplasmic phase facilitated resolution of the change in Δ_μH,_ influence of external pH on cytoplasmic pH and the lack of influence of cation fluxes of K^+^ or Na^+^ (earlier postulated salt pump – Findlay et al., 1969). Beilby and Walker (1981) demonstrated instantaneous manifestation of Cl^-^ influx by voltage-clamping the membrane PD of Cl^-^-starved *Chara* cell, challenging the cell with a range of low concentrations of Cl^-^ and recording an inward positive current (**Figure 4C**). Clamping the membrane PD prevented activation of other transporters by change in PD. The current amplitude leveled off with Michaelis–Menten kinetics (V_m_ ∼ 100 nmol/m^2^.s, K_m_ 10 – 20 μM). Beilby and Walker (1981) modeled the data with the Hill–Whittingham equation, which is appropriate for processes with low K_m_ where an unstirred layer may be important. Sanders (1980b) and Beilby and Walker (1981) agreed that Cl^-^ is co-transported with 2H^+^, with net positive charge influx.

Sanders and Hansen (1981) formulated a kinetic model, where three reaction steps occur on either surface of the plasma membrane and loaded or unloaded carrier crosses the membrane. This scheme postulates that Cl^-^ binds on and leaves first and charge crosses membrane on loaded carrier. The model addresses the main features of the symporter: (i) Michaelis–Menten kinetics, (ii) cytoplasmic Cl^-^ concentration and pH effects on V_max_ but not K_m_, and (iii) Cl^-^ concentration and pH interaction.

#### Amine Uniporter

**FIGURE 5:**
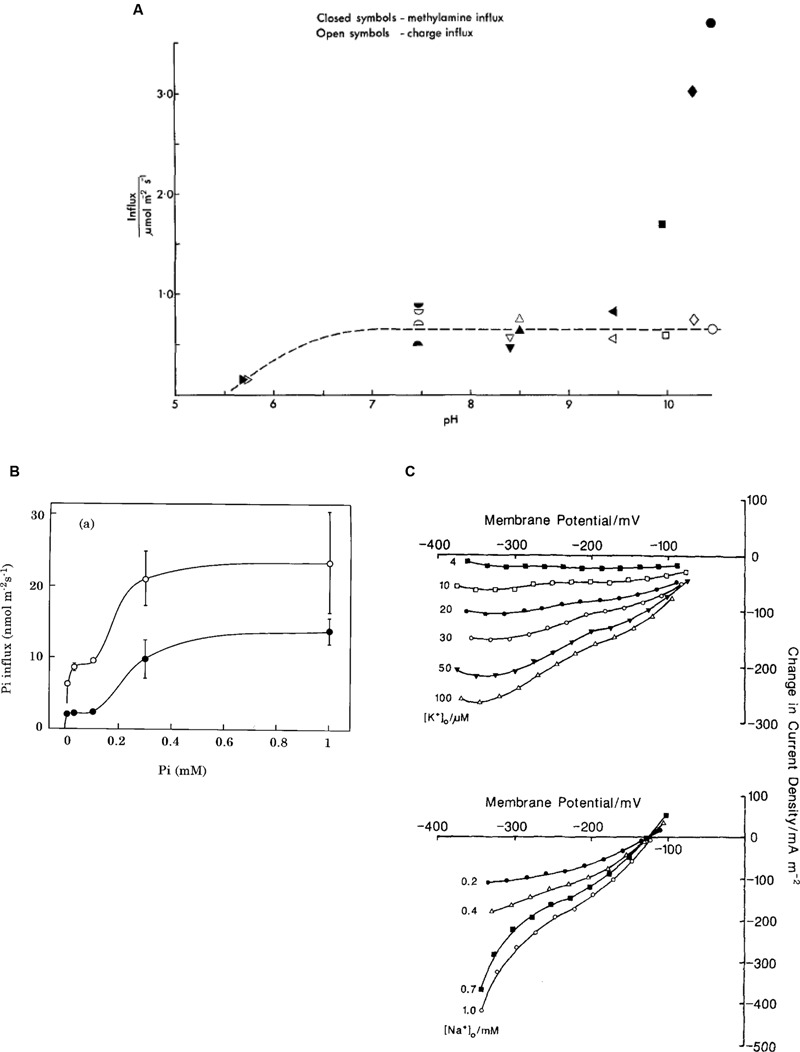
**Plasma membrane transporters.** **(A)** Amine transport: comparison of influx of charge, empty symbols, and methylamine (neutral or charged form), filled symbols (Walker et al., 1979b). **(B)** Phosphate transport: The Pi influx as function of external Pi concentration: open circles, cells pretreated without Pi for 7 days; closed circles, cells pretreated with 0.1 mM Pi for 7 days (Mimura et al., 1998). **(C)** Na^+^/K^+^ symport: Difference current-voltage curves (calculated by subtracting the 1 mM Na^+^ or 200 μM K^+^ I/V characteristics) for range of K^+^ concentrations with Na^+^ at 1 mM (top) and Na^+^ concentrations with K^+^ at 200 μM (bottom) (McCulloch et al., 1990).

Nitrogen is vital for many important plant biochemicals, such as chlorophyll, ATP, nucleic and amino acids. Smith and Walker (1978), Walker et al. (1979a,b) found that simple amines enter characean cells as cations, NH_4_^+^ and CH_3_NH_3_^+^, at pH below their respective pK_a_ of 9.25 and 10.64. Using similar technique to Cl^-^ influx investigation, Walker et al. (1979a) challenged the cell with a range of amine concentrations and recorded clamp currents, also obtaining Michaelis–Menten relationship: V_m_ up to 200 mA/m^2^, low K_m_ of ∼ 3 μM for NH_4_^+^ and 200 μM CH_3_NH_3_^+^. Clamping at different membrane PDs established exponential PD dependence of both V_m_ and K_m_. The Hill–Whittingham equation yielded unstirred layer of up to 150 μm in slow flowing media, which reduced to ∼ 40 μm in fast flowing media. The rate of transport fell rapidly with exposure to amines (At one time all Characeae cultures in the laboratory stopped reacting to amines and experiments could only be restarted after enthusiastic cleaning lady stopped mopping the lab floor with ammonium based cleaner!). The transporter was modeled as a uniport with a binding site inside the membrane and mid membrane potential energy barrier. Fairley and Walker (1987) concluded that increasingly substituted amines are transported in cationic form by the same porter. The stoichiometric ratio of the influx of charge and ^14^C methylamine was 0.9 mol/Faraday (pH 5.7 – 8.5). Above pH 9, the influx of amine increased with rising concentrations of the free bases (Walker et al., 1979b; **Figure 5A**). Ritchie (1987) measured permeabilities of ammonia, methylamine and ethylamine as P_ammonia_ = (6.4 ± 0.93) × 10^-6^ m/s, P_MA_ = (6.0 ± 0.49) × 10^-6^ m/s and P_EA_ = (8.4 ± 1.2) × 10^-6^ m/s to (14 ± 1.2) × 10^-6^ m/s. The pH_o_ in the alkaline bands is close to the pK_a_ of these amines (9.25 – 10.75), so the neutral amine transport is important. Ryan and Walker (1993) measured ammonium concentration in *Chara* vacuole of up to 70 mM, mostly in protonated form due to low vacuolar pH. To preserve electroneutrality, cells exported K^+^ and Na^+^ and imported Cl^-^ or manufactured malate in Cl^-^- free media. Ryan and Walker (1994) inhibited Glutamine synthase, enzyme involved in ammonia assimilation, by L-methionine-D, L-sulphoximine (MSX). An increased concentration of ammonia in the cell strongly inhibited amine uptake, suggesting kinetic regulation by the internal amine concentration or a concentration of an intermediate of nitrogen assimilation.

#### Phosphate Transport

Phosphorus is another vital element for building blocks of plant biochemistry: phospholipids in cell membranes, phosphate groups in DNA and RNA, ATP and other metabolic compounds in energy transduction. Inorganic phosphorus, Pi, forms phosphates: mainly H_2_${\mathrm{PO}}_{4}^{–}$ at pH 5, while ${\mathrm{HPO}}_{4}^{2–}$ dominates at pH 10. The observed Pi concentration of 5–10 mM in the cytoplasm must be maintained by active import, especially as the external concentration is in the micromolar range (Mimura, 1999). Even at this low external Pi, starvation of up to 10 days increased the influx transiently. In contrast to Cl^-^ concentration dynamics, the cytoplasmic Pi concentration did not change with starvation or when Pi was re-supplied, although the vacuolar concentration increased when Pi was available (Mimura et al., 1998). The membrane PD or the cytoplasmic pH was not affected by Pi starvation. However, the rate of Pi influx increased for up to 7 days after Pi was re-supplied (Mimura et al., 2002). If the starvation medium contained small amount of Pi (0.5–1 μM) the response was greater, but more transient. The Pi influx showed two plateaux, as the Pi concentration increased and was modeled by a low affinity transporter with K_m_ of ∼4 μM and a higher affinity transporter with K_m_ of ∼220 μM (Mimura et al., 1998; **Figure 5B**). Pi and Na^+^ uptakes were linked: Na^+^ concentration with K_m_ of 300 μM, Pi concentration with K_m_ of 10 μM. In absence of external Na^+^ induction and inactivation were abolished. Thus Na^+^ is the main cotransported ion (Mimura et al., 1998; Reid et al., 2000). Combined tracer and voltage clamp experiments established the stoichiometry of Na:Pi of 5.68 at pH_o_ 6. The stoichiometry was confirmed by voltage-clamp experiments where the influx of positive charge exceeded the influx of ^32^Pi by a factor of 6.26. The dependence of Pi influx on pH_o_ is consistent with the transported species being H_2_${\mathrm{PO}}_{4}^{–}$. Interestingly, perfused cells required ATP in the perfusion medium to reach a similar influx of Pi to that of intact cells. It is possible that hyperpolarization is necessary for this symport to function. While the electrochemical PD for H_2_${\mathrm{PO}}_{4}^{–}$ and Na^+^ suggests that there is not enough energy at pH_o_ 5, a large influx was still observed. The authors suggest that the symporter might be able to utilize H^+^ at low external pH, as observed in yeast and *Neurospora* (see Reid et al., 2000 for review).

#### Na^+^/K^+^ Transport

Similar to phosphate, K^+^ is concentrated in both cytoplasm and vacuole (up to ∼100 mM), but may be quite scarce in some ponds inhabited by freshwater Characeae. The K^+^ selective inward rectifier channels require very negative membrane PDs to open. The high conductance K^+^ channels open at less negative membrane PDs, but need external K^+^ concentrations above ∼1 mM. After the cells were starved of K^+^, Smith and Walker (1989) measured electrogenic influx of K^+^, which was dependent on Na^+^ presence in the medium. The ratio of tracer and charge inflow confirmed symport of K^+^ with Na^+^ with a stoichiometry 1:1: K^+^ with K_m_ of ∼ 30 μM and Na^+^ with K_m_ of ∼ 470 μM. McCulloch et al. (1990) observed an exponential clamp current turnoff with short half times of ∼50 s, even in low K^+^ concentrations. As K^+^ concentration in the cytoplasm is already high, Na^+^ was a more likely candidate for transport inhibition at ∼5 mM (Tazawa et al., 1974). The authors used cytoplasm-enriched fragments to overcome current turnoff and gain more time for measurements. These cell constructs are prepared by slow centrifugation of long internodal cells creating cytoplasmic plug at one end, which is then tied off by a thread. With greater volume of cytoplasm for a given surface area, it was possible to obtain families of I/V characteristics at different K^+^ and Na^+^ concentrations (see **Figure 5C**). For K^+^ influx, K_m_ decreased as the PD became more negative, while V_max_ increased. For a Na^+^ influx both K_m_ and V_max_ increased with the negative going PD. These characteristics can be modeled either by simultaneous transit of K^+^ and Na^+^, or by the ions transiting in consecutive steps. Both models suggest a double negative charge on the unloaded carrier and extracellular binding of K^+^ followed by Na^+^. The charge transit process is the limiting step at more positive membrane PDs.

#### Comparison to Land Plant Plasma Membrane Transporters

The H^+^/Cl^-^ symporter in root hair cells of *Arabidopsis* relative mustard *Sinapis alba* was described by Felle (1994). Clearly, this transporter must be active in range of tissues of land plants, as Cl^-^ compartmentation is similar to Characeae: low in the cytoplasm and high in the vacuole to maintain turgor together with K^+^ and other inorganic and organic anions (Barbier-Brygoo et al., 2000). Teakle and Tyerman (2010) lament lack of data on Cl^-^ transport under salinity stress. They point out that the electrochemical potential for Cl^-^ changes as plants are exposed to saline environment and the Cl^-^ influx might become passive through channels. The salt tolerant Characeae *Lamprothamnium* increases its vacuolar Cl^-^ concentration from 200 – 800 mM as external salinity rises (Bisson and Kirst, 1980). This is a perfect system to study the nature of Cl^-^ inflow under salinity stress.

The ammonium ion transporters belong to the AMT/MEP/Rh family found in all domains of life and described in plants and fungi in 1990s (for reviews see Ludewig et al., 2007; McDonald et al., 2011). The detailed protein structures are being investigated and suggest charged NH_4_^+^ is the transported molecule (Pantoja, 2012).

In phosphate transport, most land plants use H^+^ as the driver ion and Pi uptake causes cytoplasmic acidification (Mimura, 2001). The main family of Pi transporters are PHT1 (see Nussaume et al., 2011 for review). The Na^+^/Pi transporter in Characeae is similar to that in animal cells, which operate on Na^+^ economy. However, there are examples of Na^+^/Pi symport in some chlorophytes (Ullrich and Glasser, 1982; Ritchie et al., 1997).

Na^+^/K^+^ symport is limited to aquatic higher plants and was observed in *Egeria* and *Vallisneria* leaves, and in *Elodea* and *Egeria* roots. In land plants such as wheat, barley or *Arabidopsis*, the driver ion appears to be H^+^ (Maathuis et al., 1996). In Characeae the Na^+^ coupling is also implicated in the transport of urea, amino acids and sugars (Walker et al., 1993; Walker, 1994).

The detailed data on the control of the above transport systems by external and internal concentrations of driver and transported substances and pH were obtained utilizing the large size of characean cells and the ability to manipulate cellular compartments by perfusing the vacuole or the cytoplasm or concentrating the cytoplasm in the cytoplasm enriched cell constructs. Direct comparison of tracers and electrical currents in voltage clamped cells provided transport stoichiometries.

### Cell to Cell Transport

#### Structure of Nodal Complex

**FIGURE 6:**
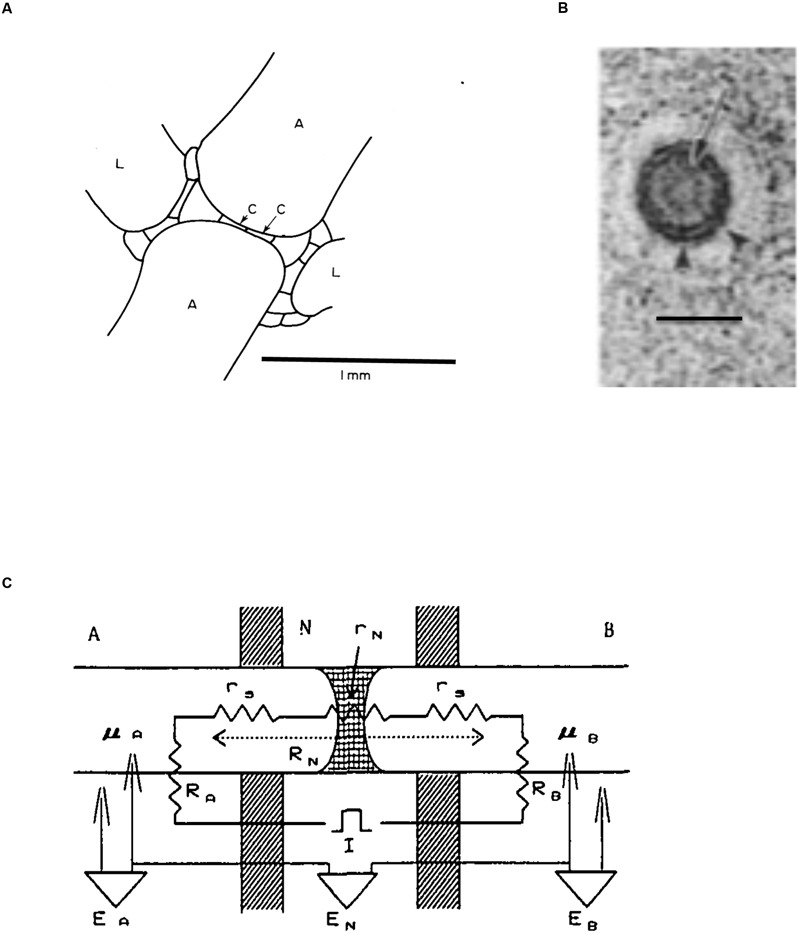
**Structure of the nodal complex. (A)** In this diagram of longitudinal section through the node of *Chara* the axial internodes are marked “A,” the lateral (leaf) internodes are marked “L” and the two central nodal cells are labeled “C.” (adapted from Walker and Bostrom, 1973). **(B)** Electron microscopy of transverse view of plasmodesma in a branch node of *C. zeylanica*. Arrow marks the central desmatubule, small arrowheads indicate the spoke structures connecting it to plasma membrane. Bar is 50 nm (from Cook et al., 1997). **(C)** The electrical model of the node and its neighboring internodes (Ding and Tazawa, 1989): R_A_ is the membrane resistance of cell A, R_B_ is the membrane resistance of cell B, R_N_ is the resistance across the node, which is the sum of the node resistance r_N_ plus the resistances of the cell sap r_s_. The PDs across cell A, cell B and the nodal region are E_A_, E_B_ and E_N_, respectively. The internal electrodes are labeled μ_A_ and μ_B_.

The axial and branch internodes are separated by nodal complexes (see **Figure 6A**, Walker and Bostrom, 1973). Shepherd and Goodwin (1992b) describe how the new internode and the nodal architecture arise from the apical cell establishing the path of cytoplasmic streaming (see Streaming). The shortest path between two internodal cells is through two flat cells in the middle of the node (marked “C” in **Figure 6A**, Spanswick and Costerton, 1967).

Transport between internodal cells is mediated, and controlled, by plasmodesmata that connect the cytoplasm of neighboring cells (for review see Burch-Smith and Zambryski, 2012). Spanswick and Costerton (1967) found that in *Nitella translucens* the plasmodesmata between the nodal cells had uniform diameter, while plasmodesmata between nodal and internodal cells developed central cavities, sometimes with several openings. Young cells exhibited extensive connectivity with up to 14.7% of the wall occupied by plasmodesmata. Characeae form primary plasmodesmata containing endoplasmic reticulum (ER) with close similarity to higher plant plasmodesmata was established (Franceschi et al., 1994; Cook et al., 1997; Brecknock et al., 2011; **Figure 6B**).

#### Fluorescent Tracers

Shepherd and Goodwin (1992a,b) attached peptides of increasing molecular weight to fluorescein to establish molecular exclusion limit in cell to cell communication: 874 Da. They used young shoots of *C. corallina* and injected fluorescein by iontophoresis, sometime aided by pressure, into the cytoplasm of one of the cells in a lateral branch. In the winter months the lateral internode cells exhibited low resting PD of ∼-120 mV and restricted cell-to-cell communication of the internode and adjacent node. As the action potential inhibited cell-to-cell communication, the exposure to excitation inhibitor La^3+^ restored communication. In spring the branch cells with more negative resting PD (∼-210 mV) increased transport of 6 carboxyfluorcescein between nodes and internodes. As in winter cells, if cytoplasmic Ca^2+^ was increased due to action potential or exposure to ionophore A23187, cell-to-cell transport was inhibited.

The spring plants became fertile and formed male reproductive structures (antheridia) from the nodal cells located near the descending (internodal) cytoplasmic stream. While the molecular exclusion limit was smaller (between 750 and 874 Da), the young antheridia were easily reached by the fluorescent dye, while the mature antheridia lost connectivity from the rest of the plant (Shepherd and Goodwin, 1992b).

Kwiatkowska (2003) combined fluorescent dye Lucifer Yellow experiments with electron microscopy to study the development of the antheridia in *C. vulgaris* and *tomentosa.* Simple plasmodesmata connect the antheridium and supporting cells in early developmental stages. Gradually some plasmodesmata disappeared enforcing radial orientation of symplasmic routes through the antheridium. Simple plasmodesmata developed branching to provide pathway for gibberellins and nutrients. Then the plasmodesmata were selectively plugged, limiting the synchronization of cell divisions. Finally the plasmodesmata between the antheridium and the thallus were spontaneously broken, starving the antheridium of gibberellins and initiating spermiogenesis. ER penetrated into antheridial filament plasmodesmata at specific stage of spermiogenesis enabling exchange of nucleohistones into nucleoprotamines. These results confirm that the plasmodesmata are very dynamic structures under tight control of the plant.

#### Radioactive or Substitution Tracers

Bostrom and Walker (1975) employed two internodal cells in tandem to measure the intercellular transport of Cl^-^. The cells were placed in three-compartment holder similar to that in **Figure 2A**. The node was positioned in the middle compartment (B) and compartments were insulated by silicone grease. The ^36^Cl tracer was added to one compartment and the content of the tracer in both cells was monitored over time. The chloride was taken up and about two thirds remained in the vacuole of the exposed cell, while a third was transported to the other internode. No polarity was found, as the results were insensitive to swapping the basal and apical internodes as input cells. The rate of transport varied between 4 and 60 pmol.s^-1^ and was consistent with diffusion through the plasmodesmata without invoking bulk flow or active transport. Bostrom and Walker (1976) controlled the speed of cytoplasmic streaming by cytochalasin B. The intercellular chloride flux was proportional to the streaming speed in the input internode but not the “sink” internode. Streaming speed did not affect the chloride influx in the input cell.

Chloride is not consumed in metabolism or complexed into compounds, but its distribution between cytoplasm and vacuole can be complicated by tonoplast action potentials elicited by handling the cells. MacRobbie (1969, 1975) initially postulated vesicle transport to explain the fast appearance of chloride tracer in the vacuole. Later she performed elegant experiments exposing only half of the internodal cell to radioactive label. A blockage of excitation diminished the fast “vesicle transport” phase.

Ding and Tazawa (1989) substituted Rb^+^ for K^+^ and exposed the input cell to 100 mM RbCl at 5°C. The low temperature inhibited the transnodal transport and 43 mM Rb^+^ accumulated in the cytoplasm. Upon temperature increase to 25°C, ∼ 12% was transported into the sink cell, suggesting diffusion process with coefficient 2.3 × 10^-11^ m^2^.s^-1^ (plasmodesmata were assumed to occupy 10% of the nodal area). The rubidium transport was also strongly dependent on cytoplasmic streaming in either or both internodes, regardless if the streaming speed was controlled by cytochalasin B or change in temperature. A turgor pressure gradient of 240 mOsm across the node decreased the nodal transport, suggesting the existence of valving system.

Zawadski and Fenson (1986a) measured intercellular transport of dissolved inorganic carbon (DIC) by supplying NaH^14^CO_3_ to the input cell. Their results were complicated by the complexity of DIC distribution in the medium as CO_2_ or bicarbonate according to pH, the intricacy of the banding system (see Beilby and Bisson, 2012) and carbon fixation by photosynthesis. In this case low streaming rate in either cell resulted in diminished intercellular transport. In the winter the transport was more sensitive to anoxia and decrease in illumination. In the summer the cells have greater reserve of ATP, so it is possible that the DIC transport is active. The application of pressure gradients on the node confirmed this hypothesis. Zawadski and Fenson (1986b) found that trans-nodal transport of ^14^C was independent of the direction of the pressure gradient (up to 2.5 bars). However, the rate of transport decreased with the increasing pressure gradient. The plasmodesmata are likely to contain pressure sensitive valving system. Similar pressure sensitivity was also found in plasmodesmata of higher plants (Oparka and Prior, 1992). Transports of ^36^Cl, ^32^P and ^42^K were also affected by imposed pressure gradients: ^42^K transport was consistent with diffusion, but active components were postulated in both ^36^Cl and ^32^P transport. Large portion of the ^32^P in the input cell was metabolized, resulting in small feed into the sink cell. Working with whole plants of *C. hispida*, Box et al. (1984) measured the flux of ^14^C from the rhizoids to the top of the plant. The rate of transport was reduced to 6% by exposure to cytochalasin B. However, transport of ^32^P was somewhat slower than cytoplasmic streaming probably due to involvement in metabolism. Trebacz et al. (1988) found that blockers of photosystem I or II, such as 3-(3,4-Dichlorophenyl)-1,1-dymethylurea (DCMU), 2,6-Dichlorophenolindophenol (DCPIP) or uncouplers of phosphorylation Iodoacetamide (IAc) 2,4-Dinitriphenol (DNP), Diethyl stilbestrol (DES), NH_4_^+^ and citrate all diminished intake of DIC and reduced the transport across nodes. These findings suggest that the transport of carbon, chloride and phosphate compounds across the node is at least partially active.

Trebacz et al. (1988) identified pentoses, hexoses and disaccharides formed from the supplied NaH^14^CO_3_ by high performance liquid chromatography in the feed cell. These and small amino acids passed through the node into the adjacent cell. Ding et al. (1992) fed NaH^14^CO_3_ to a branchlet of *C. corallina* in a internode-branchlet complex and measured photoassimilates after 10 min in both the source branchlet and the sink internode, using thin-layer chromatography. The main photoassimilates transported were sucrose and amino acids. Transport was aided by downward concentration gradients of sucrose, serine and glutamic acid between the cytoplasm of the branchlet and the internodal cell, which decreased when the apex was detached.

#### Electrical Measurements

The insertion of electrodes into the internodes on each side of the node allows measurement of transnodal PD and also the transmembrane PD of each internode (with appropriate reference electrodes in each compartment). It is also possible to pass current across the node to measure electrical resistance. The node resistance varied in different systems: ∼1.7 kΩ.cm^2^ in *Nitella* (Spanswick and Costerton, 1967), ∼ 0.47 kΩ.cm^2^ in *C. corallina* (Bostrom and Walker, 1975), 0.06 – 0.12 kΩ.cm^2^ in the nodes between young branch *Chara* cells and 0.2 – 0.51 kΩ.cm^2^ in the older branch cells (Reid and Overall, 1992). Ding and Tazawa (1989) measured the nodal resistance/conductance as a function of imposed pressure gradient. The pressure gradient of 180 mOsm diminished the conductance to ∼50%, but no further decrease was obtained with further pressure rise. The location of increased electrical resistance was found on the side of the node adjacent to the internode with reduced turgor. Thus the nodal cells deform by bulging out into the less turgid internode closing the valving mechanism. The rubidium trans-nodal flux diminished to 3% in such conditions. The authors formulated an electrical model of the node and adjacent internodes (see **Figure 6C**). In similar experiments the exposure of one of the tandem *Chara* internodes to 100 mM mannitol increased nodal resistance by 40% (Cote et al., 1987).

The metabolic inhibitor carbonyl cyanide m-chlorophenylhydrazone (CCCP) affected internodes by making the membrane PD less negative, increasing the membrane resistance and stopping the cytoplasmic streaming (Reid and Overall, 1992). The *trans*-nodal resistance greatly increased with the same time course. The exposure of one internode to 200 mM mannitol resulted in doubling of the nodal resistance. The fluxes of ^14^C butyrate and ^36^Cl were diminished both by CCCP and mannitol. The excision of one internode resulted in rapid resistance increase followed by further slow rise. The cytoplasmic pH changes induced by butyric acid, NH_4_^+^ or methylamine resulted in small effects on the trans-nodal resistance. On the other hand, the *trans*-nodal resistance was insensitive to changes in light intensity, cytoplasmic calcium concentration changes or *trans*-nodal PD manipulation.

Sibaoka and Tabata (1981) concentrated on action potential (AP) transmission across nodes of *C. braunii.* They inserted additional electrode in one of the large nodal cells. The whole nodal cell was not excitable, but the adjacent area of the nodal cell (end-membrane) displayed APs. At the time of AP conduction the stimulated internode produced greater electrotonic depolarization in the next internode without the end-membrane excitation, facilitating the internode–internode transmission. The authors modeled the internode with three resistances R_a_ (internode a), R_b_ (internode b) and nodal resistance R_n_ (compare to more complex model in **Figure 6C**). The coupling ratio for internode a was calculated as R_b_/(R_b_ + R_n_). This ratio increases if R_b_ > R_n_ or if APW (artificial pond water) in the nodal compartment was replaced by more conductive medium of 1 mM KCl or 10 mM NaCl. R_n_ was estimated as 1.2 kΩ.cm^2^.

#### Conclusion

The early measurements of intercellular transport and electrical conductance provided basis to what is now a large field mainly centered on structure and evolution of plasmodesmata (Burch-Smith and Zambryski, 2012; Evkaikina et al., 2014). In gymnosperms and angiosperms primary plasmodesmata develop at the time of cell division, while secondary plasmodesmata can form between any adjacent cells after cell division. Both primary and secondary plasmodesmata were observed in Characeae, but in different species of *Chara* (Franceschi et al., 1994; Cook et al., 1997; Brecknock et al., 2011). However, some early land plants such as Selaginellaceae and ferns seem to lack the ability to form secondary plasmodesmata (Evkaikina et al., 2014). Raven (2005) suggests complex evolutionary pathway: independent evolution of plasmodesmata in brown algae, in characean algae, and up to five times (!) in green algae, but not in red algae, haptophytes and dinoflagellates, despite multicellular morphology. In embryophytes plasmodesmata facilitate exchange of miRNA, proteins, and mRNA between adjacent cells in the course of plant development. The full role of characean plasmodesmata is yet to be investigated.

### Streaming

#### Streaming and Morphology

**FIGURE 7:**
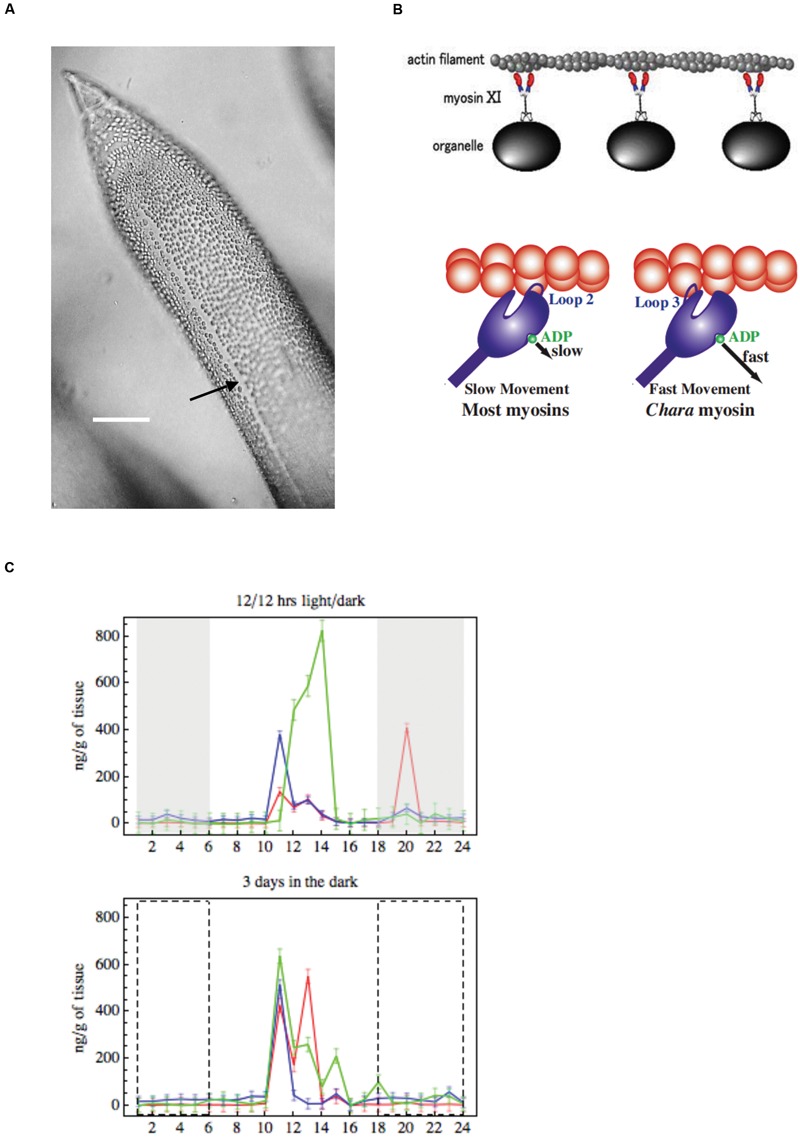
**(A)** Cytoplasmic streaming Young leaf *Chara* cell showing the neutral zone between the two opposing cytoplasmic streams (black arrow), bar 500 μm (from Beilby and Casanova, 2013). **(B)** Model of cytoplasmic streaming (Shimmen, 2007): *Chara* myosin, which mediates the fast streaming speed, contacts actin via loop 3, with fast ADP release (most other myosins use loop 2, which slows ADP release). **(C)** Endogenous concentrations of auxin, melatonin and serotonin: changes over 24 h in IAA (green), melatonin (red) and serotonin (blue) in summer *Chara* thalli. Top graph: plants sampled straight from growth tank maintained on 12/12 light/dark cycle, darkness is indicated by gray rectangles. Bottom graph: explants were pretreated 3 days in darkness, the dashed line rectangles indicate where plants experienced dark in the 12/12 h regime (adapted from Beilby et al., 2015).

The large size of characean cells makes observation of cytoplasmic streaming easy, requiring only low power microscope. The streaming pattern follows the orientation of the chloroplast rows, tracing a helical path around the cell, the upward and downward streams separated by a chloroplast-free “indifferent zone” (**Figure 7A**). As the chloroplast rows are fixed in the Characeae, the streaming direction is related to the whole plant development. The oldest leaf cell in each whorl and axillary new shoots grow below the descending cytoplasmic stream. The streams run in opposite directions on each side of the nodal complex (Hope and Walker, 1975). The velocity of streaming is comparatively steady given constant temperature and supply of ATP.

#### Mechanism: Actin and Myosin

The ribbon of cytoplasm (∼10 μm thick) winds its way around the cell with the same speed, transferring some of the movement to the vacuole. This agitation drops off with the distance into vacuolar sap. Kamiya and Kuroda (1956) prepared cytoplasm-enriched cell constructs (see Na^+^/K^+^ Transport) with greater volume of flowing cytoplasm and observed similar speed decrease further away from the gel (static cytoplasm). The authors proposed the “sliding theory,” where the interactions of sol (flowing cytoplasm) and gel surfaces produce the shearing force that moves the sol along. Using light microscopy Kamitsubo (1966) observed rope-like structures on the cytoplasmic side of the chloroplasts. Nagai and Rebhun (1966) and Kersey and Wessells (1976) employed electron microscopy to resolve about 100 microfilaments making up each rope. Kamitsubo (1972) used strong illumination to detach chloroplasts and the cables on a small patch of the cell, strongly inhibiting the streaming. When the cables regenerated, streaming was restored. Palevitz et al. (1974) and Williamson (1974) observed arrowhead structure, found in animal systems with actin, when heavy mero-myosin (HMM) sub fragment S1 is applied. Nothnagel et al. (1981) confirmed actin presence by fluorescently labeled phallotoxin, while Williamson and Toh (1979) used an antibody raised against actin. Williamson (1972) and Shimmen and Tazawa (1983) stopped streaming by applying the animal systems inhibitor of actin-based motility, cytochalasin.

In analogy to muscle movement it seemed likely that myosin also participates in cytoplasmic streaming. Kato and Tonomura (1977) purified myosin from *Nitella*. Chen and Kamiya (1975, 1981) located myosin in the cell by moving cytoplasm into one half of the cell by centrifugation. If the half without cytoplasm was treated by SH reagent *N*-ethylmaleimide (NEM), or heat of 47.5°C, the subsequent streaming was not affected. When the same treatments were applied to cytoplasm-containing half of the cell, the streaming was disrupted in the whole cell. Therefore, similar to muscle, characean myosin is more sensitive to NEM and heat than actin and is found in the cytoplasm (for references see Shimmen and Yokota, 1994). Williamson (1975) observed cytoplasmic organelles, which became strongly bound to actin cables, when ATP was removed from perfusion medium in tonoplast free cells. Using electron microscopy Nagai and Hayama (1979) observed horn-like shapes with small globules (∼30 nm diameter) on endoplasmic organelles. Kachar and Reese (1988) agreed that myosin may be found in ER, to facilitate sliding along the actin cables.

In characean cells the streaming speed can reach 100 μm/sec, much greater than that in higher plants and actin-myosin sliding in skeletal muscle. In “mix and match” experiments, glass was coated with myosin. The fluorescent-labeled actin filaments were added and their movement could be observed (Kron and Spudich, 1986). Shimmen and Yokota (1994) combined myosin from characean cytoplasm and actin filaments from skeletal muscle to observe similar sliding speeds to those in characean cytoplasmic streaming. Shimmen and Yano (1984) set up tonoplast-free cell with characean actin and perfused it with latex beads coated with skeletal muscle myosin. The slow velocity of this combination indicated that the speed of characean streaming is due to the native myosin.

Genetically characean myosin is similar to that of land plants: myosin XI. The myosin molecule was resolved by electron microscopy, showing two head structures and a tail that mediates the binding process (Shimmen, 2007, **Figure 7B**). While myosins in other species have positive charge on loop 2 and several lysine residues, *Chara* myosin loop 2 is uncharged with no lysine cluster. Instead, the loop 3 is positively charged (Ito et al., 2009), which leads to high ATPase activity. The less charge on loop 2 enables higher velocity. The structure of myosin, altered accordingly, speeded up the sliding in *Dictyostelium*.

#### Energy Source: Adenylates and Mg^2+^

Williamson (1975) and Tazawa et al. (1976) determined that ATP provides energy for the myosin head to release. Without ATP the organelles are connected to actin cables by rigor cross-bridges (Nagai and Hayama, 1979). Shimmen (1978) found that maximum streaming velocity was reached at ATP concentrations above 200 μM, well above the normal cytoplasmic concentration of 0.5 – 3.4 mM. The relationship between streaming velocity and ATP concentration is linear. Consequently, inhibitors that diminish ATP concentration also affect streaming (Reid and Walker, 1983). Reid and Walker (1983), Shimmen (1988) perfused tonoplast-free cells with cytoplasm-like medium containing 1.6 ATP, 0.6 ADP, 0.8 AMP, 14.7 Pi and 2 pyrophosphate (in mM) and obtained normal streaming speeds. If only ADP was included in the perfusion medium, it was converted to ATP by adenylate kinase and streaming started after a delay. This streaming recovery was abolished by addition of adenylate kinase inhibitor to ADP medium. If the Mg^2+^ concentration in the perfusion medium was decreased compared to that of ATP, streaming speed declined (Shimmen, 1978, 1988). Shimmen and Tazawa (1983) confirmed the importance of Mg^2+^ by including Mg^2+^ chelator in the media, inhibiting streaming in both tonoplast-free and permeabilized experimental preparations. As in muscle Mg^2+^ is necessary for myosin ATPase reaction and it also maintains the streaming system.

#### Temperature, pH, Light and Ca^2+^

Shimmen and Yoshida (1993) made detailed measurements of sensitivity of cytoplasmic streaming to temperature (for historical temperature measurements see references in Shimmen and Yokota, 1994). With pH and Ca^2+^ concentration controlled in perfused cells, there is a linear relationship between streaming velocity and temperature decrease in the range 25 – 0.5°C. Some intact cells did show a steeper drop in streaming speed between 15 and 10°C.

Fujii et al. (1979), Tazawa and Shimmen (1982) employed the tonoplast-free system to explore the effect of pH, finding the greatest streaming velocity at neutral pH. Shimmen and Tazawa (1985) investigated the effect of carboxylic acid secreted by leaf-cutting ant, myrmicacin. At low pH, the undissociated form of the carboxylic acid penetrates the plasma membrane and acidifies the neutral cytoplasm by dissociation. Thus the cytoplasmic streaming is only affected if myrmicacin is applied at low external pH. Barr and Broyer (1964) reported higher velocity of streaming upon illumination, which was abolished by photosynthesis inhibitor DCMU (Plieth and Hansen, 1992). Miller and Sanders (1987) measured a decrease in cytoplasmic calcium concentration upon illumination, possibly due to Ca^2+^ uptake by the photosynthesising chloroplasts.

The early experiments researching the effects of Ca^2+^ on streaming were done on cytoplasmic droplets, which contained rotating chloroplasts (Hayama and Tazawa, 1980). The authors assumed that same actin-myosin mechanism was involved as in cytoplasmic streaming. Iontophoretic injection of different ions into the droplet produced different effect on the chloroplast movement: K^+^ and Mg^2+^ had no effect, Ca^2+^ stopped the movement transiently, Sr^2+^ and Ba^2+^ had similar effect to Ca^2+^, Mn^2+^ and Cd^2+^ induced slow irreversible decline in motion. After Kikuyama and Tazawa (1982) stopped the streaming transiently by direct injection of CaCl_2_ into intact *Nitella* cell, Williamson (1975), Hayama et al. (1979) turned to tonoplast-free cells and found that up to 1 mM Ca^2+^ was needed to stop the streaming with incomplete recovery. Tominaga and Tazawa (1981) monitored streaming with time after perfusion and found that it became more sensitive to Ca^2+^ concentration. However, compared to intact cells the Ca^2+^ concentration for streaming stoppage was too high: the data from the perfused cells was misleading! Using aequorin Williamson and Ashley (1982) monitored the Ca^2+^ concentration in the cytoplasm of intact characean cells at the time of excitation: the peak concentration was 43 μM in *Nitella* and only 6.7 μM in *Chara*. At the time of AP, the streaming cytoplasm appears to “freeze” completely, restarting slowly after some minutes. Shimmen and Tazawa (1983) employed the permeabilized cells to confirm that only 1 – 10 μM Ca^2+^ were needed to stop the streaming. In the tonoplast-free system the native cytoplasm is removed in the perfusion process, while during permeabilization the cytoplasm is not disrupted. Consequently, it is a component of the cytoplasm that is Ca^2+^ sensitive.

Shimmen and Yano (1986) perfused cells with beads coated by skeletal muscle myosin and, like in the muscle; the movement dependence on calcium concentration was lost. Myosin in skeletal muscle has no Ca^2+^ sensitivity. Further, the incorporation of muscle troponin-tropomyosin complex into characean actin filaments actually made higher calcium concentration necessary to start streaming. Consequently, the calcium sensitivity in the intact characean cell is associated with myosin. As most of native cytoplasm is removed in rapidly perfused cells, the calcium sensitivity changed. In animal and mold systems myosin produces sliding either in phosphorylated or de-phosphorylated state. Tominaga et al. (1987) introduced phosphatase-1 into perfusion medium and abolished the streaming stoppage at high Ca^2+^ concentrations. The inhibitors of phosphatase-1, on the other hand, totally inhibited streaming. As characean myosin only promotes streaming in de-phosphorylated state, ATP-γ-S irreversibly inhibited the recovery of streaming after it was stopped by high Ca^2+^ concentration (thio-phosphorylated proteins are not de-phophorylated with phosphatases). The authors suggest that at the time of an AP the phosphatase is activated indirectly through Ca^2+^ binding to calmodulin, as Ca^2+^ concentration rises. Calmodulin inhibitors, indeed, prevent streaming recovery only following exposure to high Ca^2+^ concentration, while steady state streaming is not affected (Tominaga et al., 1985).

#### The Importance of Streaming

Cytoplasmic streaming can be observed in many eukaryotic organisms: algae, higher plants, fungi, slime molds, nematodes and flies. The cells that utilize cytoplasmic streaming tend to be larger than the usual 10–100 μm or have specialized functions (Goldstein and van de Meent, 2015). However, some cells of normal size, such as cells in stinging nettle, parenchymal cells in onion or leaf cells in *Elodea*, exhibit slow circulation streaming. Fountain streaming can be observed in root hairs and pollen tubes of various higher plants. The details of the actin-myosin driven streaming were elucidated in characean cells, because their cell compartments can be manipulated. In large celled characean thalli streaming is crucial for intercellular transport of both nutrients and organic compounds (see Radioactive or Substitution Tracers). The pH banding that aids carbon fixation does not occur without streaming. There may be further roles of streaming in cell metabolism and improving homeostasis by enhancing vacuolar mixing (Goldstein and van de Meent, 2015).

### Evolution of Hormone Auxin and its Signaling Pathways

#### Polar Auxin Transport (PAT)

In land plants growth and development is directed by auxin indole-3-acetic acid (IAA) concentration minima, maxima and gradients. Young shoots of land plants produce IAA and transport it to roots by parenchyma cells which produce auxin influx- (AUX, LAX) and eﬄux- (PIN) supporting proteins (for review see Petrasek and Friml, 2009). 1-*N*-naphthylphthalamic acid (NPA) is an efficient inhibitor of the eﬄux PIN proteins. IAA research in Characeae can elucidate some of the developmental steps in auxin signaling and metabolic pathways from origins in chlorophyte algae (De Smet et al., 2011) to the complexity of extant land plants.

Hori et al. (2014) detected auxin in the basal branching charophyte *Klebsormidium.* In Characeae with more complex morphology, effects of external IAA and its transport through the thallus and rhizoids were investigated. Klambt et al. (1992) observed rhizoids developing in cuttings of *Chara globularis*. The polar growth of rhizoids was inhibited by explant decapitation or by addition of NPA. If IAA was added to the medium, the inhibition of growth was reversed. ^14^C IAA was retained by the explants more strongly after treatment with NPA. Thus NPA seems to inhibit IAA eﬄux as it does in higher plants. The rhizoid development in mosses is also affected by IAA (Eklund et al., 2010). Clabeaux and Bisson (2009) decapitated *C. australis* explants or tied the second internode with a silk thread to prevent basipetal transport through streaming. Greater number of axillary branches was observed in decapitated explants and below the tied thread: clear demonstration of apical dominance. However, unlike higher plants, the tied explants also produced greater number of rhizoids and addition of IAA to the medium had no effect.

Boot et al. (2012) placed one or two adjacent internodes of *C. corallina* in three-compartment chamber. The middle chamber was labeled by addition of ^3^H-IAA and the appearance of the label was then monitored in the outer chambers. After 1 hr the shoot to rhizoid directed transport of IAA was 50-times greater than that in the opposite direction. The polarity was lost upon exposure to NPA. Initially, the IAA transport through the thallus was attributed to cytoplasmic streaming, as the rate was comparable. When no rate decrease resulted from streaming inhibition by cytochalasin, Raven (2013) proposed involvement of other mechano-chemical motors such as dynein-tubulin or kinesin-tubulin. If the label was added to one of the outer compartments, large amount of IAA was detected in the middle compartment. The cortication of *C. vulgaris* prevented some of the leakage. The authors concluded that the auxin influx carrier proteins of higher plants are probably lacking in Characeae. Bennett et al. (2014) made a detailed study of PIN protein evolution, finding that charophyte *Klebsormidium* PIN structure was substantially different to that of higher plants. Thus the IAA transport proteins evolved to their present forms in different types of tissues of land plants.

#### Circadian and Seasonal Effects on IAA and Melatonin/Serotonin Metabolic Pathways

Beilby et al. (2015) measured circadian concentrations of IAA, melatonin and serotonin in *C. australis* plants. The plants, which experienced summer day-length and temperatures, exhibited distinct concentration maxima about 4 h after subjective daybreak. Similar concentration distribution persisted in plants pre-treated for 3 days in the dark, confirming a circadian rhythm (**Figure 7C**). Plants pre-treated 3 days in the light exhibited more IAA concentration maxima, while melatonin and serotonin exhibited smaller concentrations changes, less synchronized with those of IAA. In the winter plants exhibited much smaller IAA concentration maxima in the subjective dark phase, which again persisted after dark pre-treatment. Melatonin and serotonin concentrations were also much smaller, compared to summer cells, with only a weak correlation to IAA concentration changes. The close synchronization between IAA and serotonin circadian cycling suggests IAA biosynthesis by the tryptamine pathway, which intersects with the serotonin/melatonin pathway (Mano and Nemoto, 2012; Ljung, 2013). The IAA synthesis was recently investigated in charophytes by searching for sequences of tryptophan aminotransferase (TAA) and flavin monooxygenase (YUCCA) enzymes that mediate the main synthesis pathway in model plant *Arabidopsis.* Wang et al. (2014) found homologs of these enzymes in *Klebsormidium* and two Characeae, but Turnaev et al. (2015) argued that the differences are too large for the enzymes to be functional. Ke et al. (2015) suggested that the results are inconclusive. So, this is clearly a very active research area!

The data in this section indicate that polar auxin transport and circadian influence on IAA pre-dates emergence of plants on land. The advantage of using characean thalli for biochemical assays are (i) small number of large internodal cells (with their contents dominating over much smaller nodal complexes) in each sample and (ii) relatively small differentiation between axial and leaf internodes compared to variety of tissues encountered in vascular land plants. The seasonal and circadian nature of endogenous IAA concentration also highlights the importance of collecting plant samples in the right season and at the right part of day cycle.

## Conclusion

The size of characean cells provides the experimentalist with many options not available in typical plant cells or tissue. Due to this unusual morphology, Characeae were initially regarded as “interesting,” but not representative of higher plants. In recent decades, however, many higher plant-like properties are starting to emerge. The different aspects of the Characeae research are now coming together: electrophysiology, nutrient acquisition, cell to cell transport, carbon concentrating mechanisms, cytoplasmic streaming, geotropism, metabolic pathways, circadian rhythms, plant evolution, wound healing, cytoskeleton organization, cell walls, phytoremediation, lake ecology – too many topics to be discussed in this review. On the other hand, the exceptional Characeae morphology is providing insights into physical limits of cell size, transport of nutrients, homeostasis and macromolecular targeting (Goldstein and van de Meent, 2015). The Characeae system is about to become even more valuable with sequencing of *C. braunii*.

## Author Contributions

The author confirms being the sole contributor of this work and approved it for publication.

## Conflict of Interest Statement

The author declares that the research was conducted in the absence of any commercial or financial relationships that could be construed as a potential conflict of interest.
